# Activating dopamine D2 receptors reduces brown adipose tissue thermogenesis induced by psychological stress and by activation of the lateral habenula

**DOI:** 10.1038/s41598-019-56125-3

**Published:** 2019-12-20

**Authors:** Mariana Brizuela, Anna Antipov, William W. Blessing, Youichirou Ootsuka

**Affiliations:** Centre for Neuroscience, College of Medicine and Public Health, Flinders University, Adelaide, SA Australia

**Keywords:** Emotion, Physiology

## Abstract

Emotional hyperthermia is the increase in body temperature that occurs as a response to an animal detecting a salient, survival-relevant stimulus. Brown adipose tissue (BAT) thermogenesis, controlled via its sympathetic innervation, contributes to this temperature increase. Here, we have used an intruder rat experimental model to determine whether quinpirole-mediated activation of dopamine D_2_ receptors attenuates emotional hyperthermia in conscious rats. In anesthetized rats, we determined whether systemic quinpirole reduces BAT nerve discharge induced by activation of the medullary raphé and the lateral habenula (LHb). We measured BAT and body temperature with chronically implanted thermistors in conscious, freely moving, individually housed, male rats (resident rats). Either vehicle or quinpirole was administered, intraperitoneally, to the resident rat 30 min before introduction of a caged intruder rat. Quinpirole, in a dose-dependent manner, reduced intruder-elicited increases in BAT and body temperature. Pre-treatment with the D_2_ antagonist spiperone, but not the selective D_1_ antagonist SCH-23390, prevented this quinpirole-elicited decrease. In anesthetized rats, quinpirole abolished BAT sympathetic nerve discharge elicited by bicuculline-mediated activation of the LHb, but not the medullary raphé. Thus, activation of dopamine D_2_ receptors reduces the BAT thermogenesis that contributes to emotional hyperthermia. We provide evidence that these dopamine D_2_ receptors are located in the thermogenic pathway between the LHb and the lower brainstem pre-sympathetic control centre in the medullary raphé.

## Introduction

Salient, life-relevant, environmental events generate a rise in body temperature; a process referred to as psychological, stress-induced, or emotional (our preferred term) hyperthermia^1–4^. Both the vasoconstriction of thermoregulatory beds and the increased heat production in brown adipose tissue (BAT) contribute to this rise in body temperature^5^. We use an experimental model of emotional hyperthermia in which a conscious, chronically instrumented freely-moving rat (resident rat) is suddenly confronted with a second, caged, rat (intruder rat). Since there is no actual contact between the animals, the autonomic responses induced in the resident rat result from emotional, rather than physical, factors. BAT thermogenesis makes a substantial contribution to the resultant hyperthermia in our model, as well as in other social stress models^6–9^.

A body of evidence supports the view that dopamine is one of the neurotransmitters involved in thermoregulatory control regulated by the CNS. Dopamine D_2_ receptor *agonists*, administered systemically, intraventricularly or intracerebrally, decrease metabolic rate and increase heat dissipation via cutaneous circulation, resulting in the fall of body temperature^10–14^. Quinpirole, a selective dopamine D_2_ receptor agonist, that crosses the blood brain barrier^15,16^, has facilitated the interpretation of studies investigating the thermoregulatory effects of dopamine. Our laboratory was the first to report that cold-induced BAT thermogenesis in rats is substantially reduced by the systemic administration of quinpirole^17^. Further, quinpirole-mediated inhibition of BAT thermogenesis was blocked by pre-treatment with dopamine D_2_ receptor antagonists that cross the blood-brain barrier, but not by domperidone, a peripherally-acting dopamine D_2_ receptor antagonist. By directly recording BAT sympathetic nerve activity in anesthetized rats, we confirmed that quinpirole reduces sympathetic outflow to BAT; thus, implying that the drug acts within the CNS.

The lateral habenula nucleus (LHb) is involved in emotional hyperthermia. Lesioning the LHb attenuates BAT thermogenesis that contributes to emotional hyperthermia in conscious unrestrained rats, whilst stimulation of the LHb increases BAT sympathetic discharge, and BAT thermogenesis, in anaesthetized rats^18,19^. Our recent study^20^ suggests that the LHb-elicited BAT response is mediated via an indirect pathway, that includes the ventral tegmental area (VTA), to the medullary raphé - the key lower brainstem, pre-sympathetic, thermoregulatory control centre^21^. The VTA is part of the mesolimbic dopamine pathway, which is closely associated with emotional behaviour. LHb neurons, activated by aversive stimuli, provide inhibitory control over dopamine neurons in the VTA^22–24^. Therefore, it is possible that the VTA dopamine system is involved in the regulation of emotional hyperthermia.

In the present study, we first determined whether pre-treatment with quinpirole inhibits emotionally induced BAT thermogenesis in our intruder rat model. We then tested for dopamine receptor specificity by administering a low dose of spiperone (D_2_ receptor antagonist) or SCH-23390 (D_1_ receptor antagonist) prior to administration of quinpirole. In anesthetized rats, we then tested our hypothesis that quinpirole acts within the pathway linking the LHb to the medullary raphé. We activated BAT sympathetic discharge through focal microinjections of bicuculline into either the LHb or the medullary raphé, and determined whether the consequent increase in sympathetic discharge was reduced by systemic administration of quinpirole. We hypothesized that quinpirole would reduce BAT sympathetic discharge initiated by LHb stimulation, but would not affect discharge initiated by the medullary raphé.

## Experimental Procedures

### Ethics approval

All experiments were conducted at Flinders University in accordance with the Australian code for the care and use of animals for scientific purposes (8^th^ edition) and with ethical approval from the Flinders University Animal Welfare Committee. Rats were bred at the Flinders University animal facility. Animals were maintained at 20 °C, on a 12 hour light/dark cycle with access to food and water *ad libitum*. Studies were performed in male Sprague-Dawley rats (300–500 g). Flinders University ethics approval number 940/17.

### Surgical procedure for conscious rats

All preparatory surgical procedures were performed under general anaesthesia (2% isoflurane (Veterinary Companies of Australia, Kings Park, NSW, Australia) in 100% oxygen). Temperature probes were manually made from thermistors (NTH5G10P, Mu- rata, Kyoto, Japan) and sealed with silicone (RTV 3–1744, Dow Corning, Midland, MI, USA). The thermistor probes were implanted into the anterior mediastinum, ventral to the trachea (body temperature) and into the interscapular BAT region near the vein of Sulzer (BAT temperature)^6,25^. Insulated wires from each of the temperature probes were then subcutaneously passed to the head of the rat, attached to a head socket that was screwed to the skull, and secured with dental cement. For drug administration, a catheter was implanted into the intraperitoneal cavity, and was also passed subcutaneously to the head where it was attached to the head socket with dental cement. Following surgery, analgesia (Carprofen 5 mg/kg s.c.) (Norbrook Laboratories, Melbourne Australia) and antibiotics (Baytril 0.1 ml s.c.) (Bayer Australia, Pymble, NSW, Australia) were administered. Once the rat had fully recovered from anaesthesia, it was individually caged and returned to the animal holding room for a recovery period of at least one-week. Once all experimental recordings were completed, the animal was euthanized with pentobarbitone sodium (180 mg/kg i.p.) (Virbac Pty Limited, Milperra, NSW, Australia).

### Recording of physiological parameters, drug administration and the intruder-stress model

One day prior to the first experiment, the resident rat was placed into a plastic, open roofed, ‘home cage’ (350 mm wide × 400 mm long × 450 mm height) located in a temperature-controlled recording chamber (Biomedical Engineering, Flinders University). The ambient temperature in the chamber was maintained at 26 °C. The food container was suspended from a high-frequency response strain gauge to allow for the observation of timing of each meal and the amount consumed^26^.

The head socket was connected to a temperature recording device via a flexible cable and counter-balanced swivel device (SL12C, PlasticOne, Roanoka, VA, USA) that was located above the home cage. BAT and body temperature signals were passed to a bridge amplifier (Biomedical Engineering, Flinders University) and digitized (1 Hz) with PowerLab (ADInstruments, Castle Hill, NSW, Australia). Behavioural locomotor activity of the resident rat was recorded with a pyroelectric passive infrared sensor (NaPiOn, AMN1111, Panasonic, Osaka, Japan) positioned above the home cage. Behavioural activity signals were digitized at 100 Hz and expressed as the total amount of active time (sec) per minute (sec/min). The implanted catheter was connected to tubing that was accessible from outside the recording chamber, allowing for drug administration with minimal interference to the animal. The rat was kept in the home cage for the entire experimental period. All experimental procedures were conducted during the dark period.

Prior to experimentation, each resident rat displayed typical ultradian increases in BAT and body temperature, with episodes occurring at approximately 1–2 hour intervals^25^. Observation of these rhythms facilitated the selection of a suitable baseline to be used in the experimental study. All drugs were administered between ultradian episodes when BAT temperature was near the baseline value for each individual animal.

Thirty minutes after vehicle (Ringer) or quinpirole (5, 25, 250 µg/kg) administration, the chamber was opened and a second male rat (intruder rat) confined to a small plastic and wire mesh cage (19 × 29 × 12 cm) was introduced to the home cage. Recording of the experimental parameters continued throughout the 30-min intrusion period.

**Figure 1:**
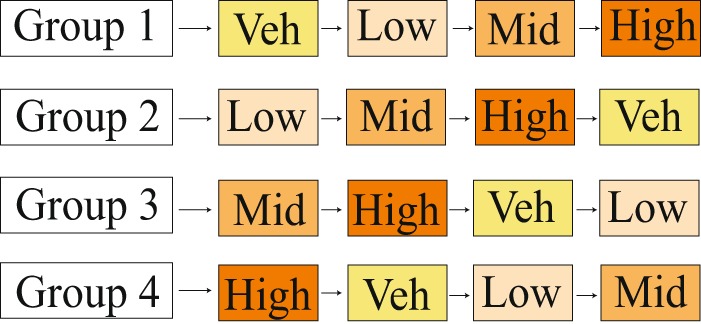
Counterbalanced design of intraperitoneal quinpirole injection for conscious experiments in rats. A rotating order of injections was used to control for serial drug effects, allowing a three-day period between each injection. Veh (vehicle was Ringer solution), Low (lowest concentration of the drug used was 5 µg/kg), Mid (mid range concertation of the drug was 25 µg/kg), High (highest concentration of the drug was 250 µg/kg).

For the second experimental section, either spiperone (0.2 μg/kg), R(+)-SCH-23390 hydrochloride (100 µg/kg), or vehicle was administered intraperitoneally. Thirty minutes following drug or vehicle administration, a second drug injection (quinpirole, 250 µg/kg) was given. After a further 30 mins, the intruder test was conducted as described above. Each rat received no more than five injections, in a counterbalanced rotating order to control for serial effects, with three days between each injection (Fig. 1)^27,28^.

### Anaesthetized experiments

Surgery was performed as previously described^20^. Briefly, rats were anesthetized with isoflurane (2% in oxygen) and an endotracheal tube was inserted via a tracheotomy. The right femoral vein and artery were cannulated for the injection of drugs and measurement of arterial pressure, respectively. Rats were later anesthetized with a mixture of urethane (400–800 mg/kg) and alpha-chloralose (40–80 mg/kg) after isoflurane anaesthesia was discontinued. Rats were mounted onto a stereotaxic frame, the bregma-lambda plane was aligned for a skull-flat configuration and a burr-hole craniotomy was made to allow for chemical injection over the LHb (3.6 mm caudal from Bregma, 0.7 mm lateral from midline, 4.6 mm deep from the cortex surface) or the rostral medullary raphé (12 mm caudal from Bregma, midline, 9.2 mm deep from the cortex surface). Rats were subsequently paralysed with d-tubocurarine (0.3 ml i.v. of a 0.3 mg/ml solution in Ringer) and artificially ventilated with 100% O_2_ (60–65 cycle/min, volume 2–3 ml/cycle). Animals were allowed to recover from paralysis between doses, so that adequate anaesthesia could be confirmed before paralysis was re-established. Body and BAT temperature were measured with thermocouples (TC-2000; Sable Systems). Body temperature was maintained with a water jacket^26^. End expiratory CO_2_ (Normocap; Datex, Helsinki, Finland) was directly recorded as per previous studies^18,27^ and maintained at 4–5% in resting condition.

An interscapular BAT sympathetic nerve was isolated as described previously^29^. Nerve activity was recorded using a pair of silver electrodes, amplified (gain 20,000, NL104 amplifier, Digitimer Ltd., Welwyn Garden City, U.K.) and filtered (band-pass 1–1000 Hz, NL125 filter, Digitimer). At the end of experiments, a ganglionic blocker, chlorisondamine chloride (10 mg/kg i.v.), was administered to confirm the loss of BAT sympathetic nerve activity and ensure that the nerve recording was made from postganglionic sympathetic axons. All data was sampled and digitized by PowerLab at 1 kHz. The amplitude of BAT sympathetic nerve discharge was expressed as a total power spectral (between 0–20 Hz, 5.12-s segment)^27^. The total power was shown in decibel microvolt (10 × log (power), dBμV)^27^.

Bilateral injections were made in the LHb (n = 6) or in the rostral medullary raphé (n = 8) using a long-shanked 5-µl glass micropipette filled with bicuculline ((−)-bicuculline methiodide, TOCRIS) (1nmol, 100 nl). Fluorescent beads (FluorSpheres carboxylate-modified microspheres, Molecular Probes) were added to the drug before injection to aid in the histological examination of injection sites. Intravenous injections of Quinpirole (25 µg/kg) were made after bicuculline ((−)-bicuculline methiodide) injection into either the LHb or the rostral medullary raphé.

### Histological confirmation of brain injection sites

At the end of the experiments, animals were perfused transcardially with washing solution (140 mM NaNO_2_, 1 mM phosphate buffer pH 7.4; 0.2 mM NaH_2_PO_4_.H_2_O, 0.8 mM NaH_2_PO_4_) followed by formaldehyde-fixatives (4%). The brains were removed for histological confirmation of injection sites via visualization of fluorescence. Serial sections (50 µm) were cut with a cryostat (Leica biosystems). Sections were visualized and imaged immediately after mounting using an Invitrogen EVOS FL fluorescent microscope (Thermo Fisher Scientific). Images were superimposed using ImageJ freeware.

### Data analysis

Physiological signals were recorded with PowerLab (AD Instrumets) and analysed with Igor Pro (WaveMetrics, Portland, OR). Statistical analysis was performed using SPSS (IBM Corp, version 25) and Graph-pad Prism 6 (Graph-pad software Inc., CA). Dose-related responses for quinpirole administration were assessed using linear regression between log-dose and the measured parameters. If the regression was not significant, factorial ANOVA was then used to determine if the parameter was different from other experimental conditions.

In the conscious animal study, to evaluate the effect of drug administration on baseline BAT and body temperature, the difference between the mean of the 5 min period prior to drug administration and the mean of the 5 min period at 23–28 min post-drug administration was calculated (delta BAT and body temperatures from the pre-injection level) as described previously^27^. To determine the effects of the intruder-stress for each experimental condition, the change in BAT and body temperature from the 10 min period prior to intruder introduction and temperature values at 18–28 min following intruder introduction were observed (delta BAT and body temperature from the pre-intruder level). Due to inter-animal variation, particularly in regards to the onset of the intruder-elicited temperature increase, this longer time period was selected^27^. For behavioural activity, the mean activity value was calculated as a percentage of the first 5 min after injection or intruder introduction^27^. For each experimental variable and drug combination examined in the intruder situation, factorial ANOVA was also used to determine whether the values observed were significantly different from other experimental groups. The Fisher’s least significant difference test was used for post-hoc analysis. Significance level was set as *P* < 0.05 for all statistical evaluations. In the conscious study, the number of recordings between recorded parameters (BAT and body temperature, and behavioural activity) was not the same in some cases due to signal failure in the probes. Group results (mean ± SEM) were calculated for each experimental condition.

### Drugs

Quinpirole hydrochloride (Sigma-Aldrich, St. Louis, USA), spiperone (Sigma-Aldrich) and R(+)-SCH-23390 hydrochloride (Sigma-Aldrich) were dissolved in 0.5 ml Ringer solution. Doses for spiperone and SCH-23390 were obtained from our previous reports^28^.

## Results

### Studies in conscious rats

#### Effect of quinpirole on intruder-elicited increases in BAT and body temperatures, and behavioural activity

**Figure 2:**
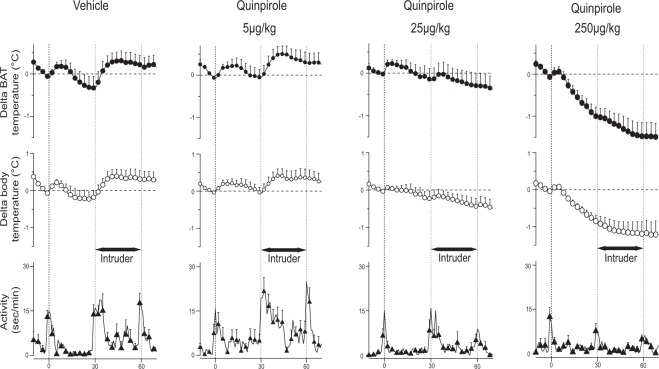
Group data (mean ± SEM) from the quinpirole dose-response experiment. Averaged experimental records from the resident rats after vehicle (n = 7) or quinpirole (n = 6 for each group) treatment showing BAT and body temperatures, and behavioural activity. The administration (time 0) was made 30 min before the introduction of the intruder rat. The intruder was introduced for 30 min (horizontal black bar with arrows).

**Figure 3:**
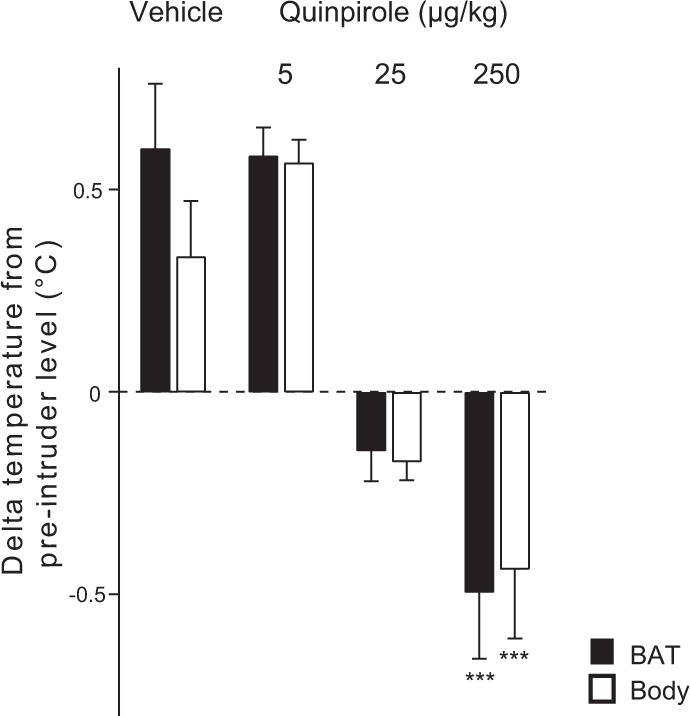
Group results (mean ± SEM) of the effect of vehicle or quinpirole administration on intruder-elicited changes in BAT and body temperature. Asterisks indicate significant linear regression between log-dose of quinpirole and changes in BAT and body temperatures. ***P < 0.001.

Both BAT and body temperatures rapidly increased after introduction of the caged intruder following pre-treatment with vehicle (Fig. 2). Quinpirole significantly attenuated the intruder-elicited increases in the temperatures, in a dose-related manner (log-dose regression for delta BAT temperature, *F*_1,16_ = 36.651, *R*^2^ = 0.70, *P* < 0.001; for delta body temperature, *F*_1,16_ = 31.043, *R*^2^ = 0.66, *P* < 0.001) (Fig. 3). The middle dose (25 μg/kg) inhibited the intruder-elicited increases in BAT and body temperature, without affecting the baseline temperature. For the lowest dose of quinpirole (5 μg/kg), the intruder-elicited temperature responses were not different from those in the vehicle group (*P* > 0.05).

When the caged intruder was placed into the home cage, the resident rat with vehicle pre-treatment became very active, circling and climbing on top of the intruder’s cage, as previously described^6^. Quinpirole dose-dependently reduced the resident’s rat activity levels following introduction of the intruder (*F*_1,16_ = 12.641, *R*^2^ = 0.44, *P* < 0.01) (Fig. 2).

#### Effects of pre-treatment with the dopamine D_1_ antagonist SCH-23390 and the dopamine D_2_ antagonist spiperone on quinpirole’s actions in the intruder rat model

**Figure 4:**
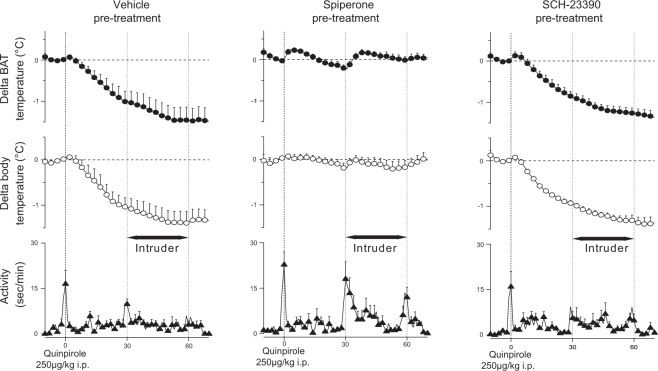
Group data (mean ± SEM) from dopamine receptor antagonist experiments. Averaged experimental records from resident rats for quinpirole (250 µg/kg i.p.) following pre-treatment with vehicle (n = 7), spiperone (0.2 µg/kg, i.p.) (n = 8) or SCH-23390 (100 µg/kg i.p.) (n = 7) showing BAT and body temperatures and behavioural activity. Administration of quinpirole was made 30 min after pre-treatment. The intruder rat was then introduced into the cage of the resident rat (horizontal black bar with arrows) 30 min after quinpirole administration.

**Figure 5:**
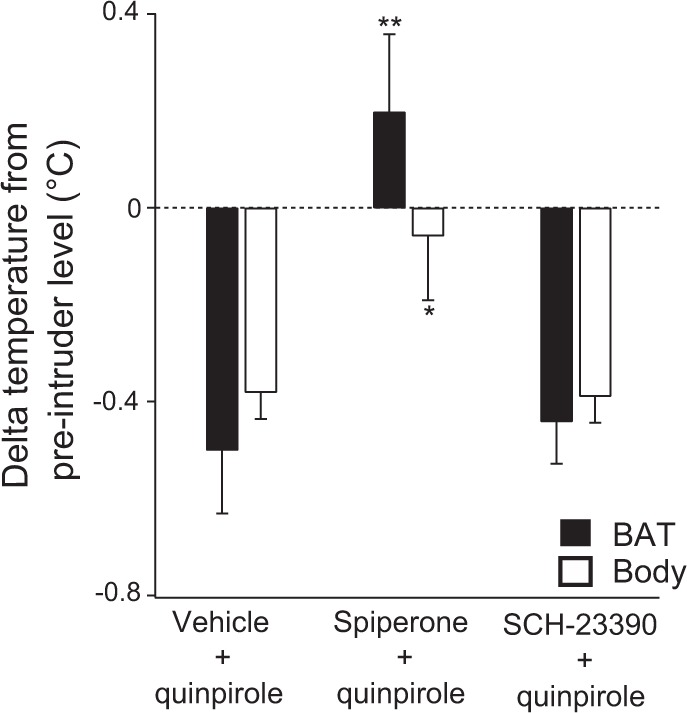
Group results (mean ± SEM) of the effect of quinpirole (250 µg/kg i.p.) following pre-treatment with vehicle, spiperone (0.2 µg/kg i.p.) or SCH-23390 (100 µg/kg i.p.) on intruder-elicited changes in BAT and body temperatures. Asterisks indicate results significantly different from vehicle pre-treatment group, *P < 0.05, **P < 0.01.

To confirm that quinpirole’s actions were mediated selectively by D_2_, and not by D_1_ receptors, the drug effects were evaluated after the pre-treatment with D_2_ or D_1_ antagonists. Data for quinpirole after pre-treatment with spiperone or SCH-23390 were compared with vehicle pre-treatment using factorial ANOVA. This comparison for BAT and body temperature was significant (*F*_2,16_ = 8.789, *P* < 0.01 for delta-BAT temperature from pre-intruder level; *F*_2, 16_ = 4.138, *P* < 0.05 for delta-body temperature) (Fig. 4). Pre-treatment with spiperone, but not with SCH-23390, prevented the inhibitory effects of quinpirole on the intruder-elicited increases in BAT (*P* < 0.01) and body (*P* < 0.05) temperatures (Fig. 5). Neither spiperone nor SCH-23390 prevented the inhibitory effect of quinpirole on intruder-elicited increases in behavioural activity (*F*_2, 20_ = 1.690, *P* > 0.05).

### Studies in anesthetized rats

#### Effects of quinpirole on BAT sympathetic nerve discharge induced by activation of the medullary raphé

**Figure 6:**
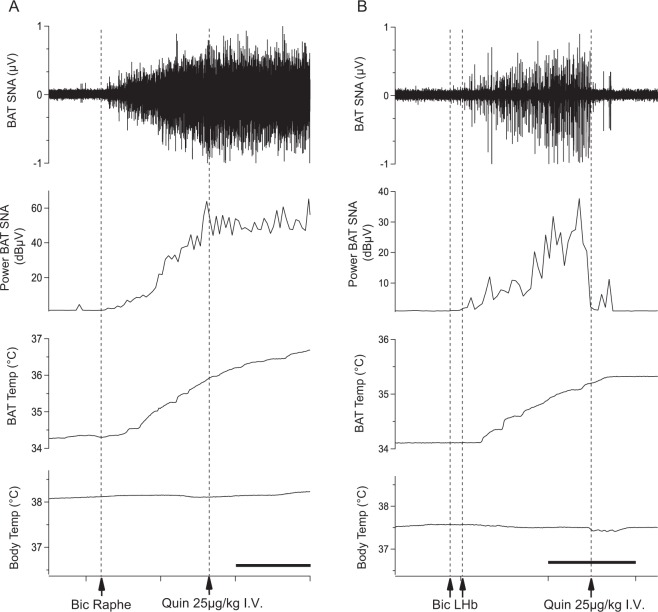
Effect of quinpirole injection (25 µg/kg i.v.) on autonomic responses elicited by injections of bicuculline (1nmol in 100 nl, Bic) into the medullary raphe (**A**) or the LHb (**B**). Chart records of simultaneously recorded brown adipose tissue sympathetic nerve discharge (BAT SNA), BAT temperature (BAT temp, °C) and body temperature (Body temp, °C) after bicuculline injection into the medullary raphe (**A**) or the LHb (**B**), and later injection of quinpirole i.v. Scale bar = 2 min.

**Figure 7:**
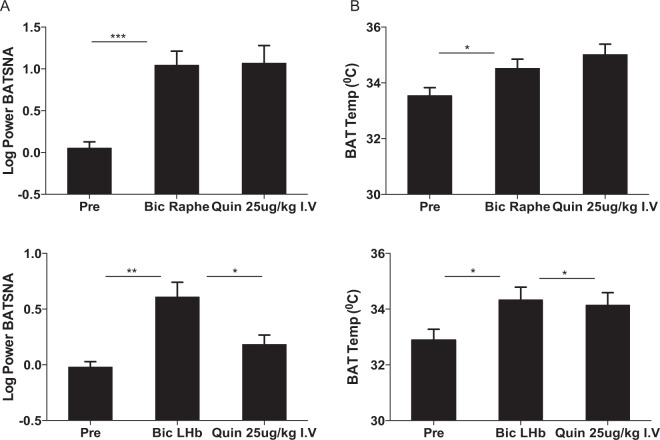
Quantitative analysis of drug effects after bicuculline injection into the medullary raphé injection or the LHb, and subsequent quinpirole injection (i.v.). Bar graphs represent maximum values after drug injections of BAT SNA (**A**) and BAT temp (**B**). Mean ± SEM, *P < 0.05, **P < 0.01, ***P < 0.001.

Under resting conditions in anesthetized rats, nanoinjections of bicuculline (1nmol in 100 nl) into the medullary raphé increased BAT sympathetic nerve activity from 1.24 ± 0.19 to 17.35 ± 5.70 dBμV (n = 8, *F*_1,7_ = 36.82 *P* < 0.001, repeated measures ANOVA) and BAT temperature from 33.5 ± 0.2 to 34.5 ± 0.3 °C (*F*_1,9_ = 21.47 *P* < 0.001, repeated measures ANOVA) (Fig. 6A). Quinpirole (25 µg/kg, i.v.) was administered after activation of the raphé. This dose was used since it sufficiently inhibited the intruder-elicited BAT thermogenesis in conscious studies. Injections of quinpirole had no effect on sympathetic nerve discharge or BAT temperature (n = 8, *P* > 0.05, Figs. 6 and 7).

#### Effects of quinpirole on BAT sympathetic nerve discharge induced by activation of the LHb

Bilateral nanoinjections of bicuculline (1nmol in 100 nl) were made into the LHb (Fig. 6). Bicuculline injections increased BAT nerve discharge from 0.98 ± 0.12 to 5.11 ± 1.54 dBμV (n = 6, *F*_1,7_ = 19.08 *P* < 0.001, repeated measures ANOVA) and BAT temperature (n = 6, *F*_1,7_ = 17.03 *P* < 0.05, repeated measures ANOVA) (Fig. 6). Quinpirole (25 µg/kg, i.v.) was administered after activation of the LHb. Quinpirole decreased sympathetic nerve discharge (1.68 ± 0.35 dBμV, n = 6, *P* < 0.05, Figs. 6 and 7A) and BAT temperature (n = 6, *P* < 0.05, Figs. 6 and 7B) to values similar to pre-injection level.

## Discussion

Our experiments provide the first demonstration that activation of dopamine D_2_ receptors robustly, and powerfully, inhibit emotionally-elicited BAT thermogenesis in a dose-dependent manner. The moderate dose of quinpirole (25 µg/kg) strongly prevented the induced temperature increases, without significant effect on resting temperatures. The behavioural activity response to the intruder animal was also reduced by quinpirole, but the response remained reasonably vigorous after the moderate dose. Thus, it is not likely that quinpirole’s inhibitory effect on BAT thermogenesis is secondary due to the general, sedative-like, effects of quinpirole. Our study confirms that quinpirole inhibits the contribution of BAT thermogenesis to emotional hyperthermia, via the agent’s selective effects on dopamine D_2_ receptors. Thus, the resulting inhibition was prevented by prior treatment with low dose spiperone, but not by pre-treatment with SCH-23390. The results are consistent with a series of papers from our laboratory^17,28^. Quinpirole’s inhibitory effect on behavioural activity was much less marked than its action on BAT thermogenesis, and the inhibition that was observed was not prevented by pre-treatment with the dopamine D_2_ antagonist spiperone; suggesting that quinpirole’s effects on behavioural activity might be mediated by presently unknown mechanisms.

Results from the anesthetized animal experiments show that quinpirole abolishes the BAT thermogenesis induced by the activation of neurons in the LHb, but not the disinhibition of neurons in the medullary raphé- strongly suggesting that quinpirole acts on CNS neurons located in the thermoregulatory circuitry linking the LHb to the medullary raphé^18,19^.

The LHb is powerfully activated by negative salient events. Output from the LHb robustly activates BAT thermogenesis in anesthetized rats, and lesioning the LHb reduces emotional BAT thermogenesis in our conscious resident rat intruder model^18,19^. The LHb has no direct projection to the medullary raphé, and therefore, we suggest that the linking CNS pathway may include the ventral tegmental area (VTA).

The rostrally-projecting axons of VTA dopamine neurons constitute the mesolimbic and mesocortical dopamine systems - key components of CNS regulation of emotional function^30^. Inhibiting all VTA neurons with focal microinjections of muscimol powerfully activates BAT sympathetic nerve activity and BAT thermogenesis^20^. The LHb, and outputs from the LHb, activate GABAergic neurons in the “tail” of the VTA in order to inhibit dopamine neurons within the VTA; thereby acting as a dopamine “brake” and reducing activity in the mesolimbic reward system^22,23,31,32^. This facilitates the redirection of attention to life-relevant events in the external environment, with the presence of associated autonomic responses, including increases in body temperature (emotional hyperthermia).

Transgenic mice with reduced dopamine synthesis, including synthesis in VTA dopamine neurons, have increased energy expenditure and increased BAT thermogenesis^33^. Moreover, loss of D_2_ receptors leads to increased energy expenditure and decreased body weight in experimental animals^34^, suggesting that increased BAT thermogenesis might contribute to this weight loss. It may be the case that activation of dopamine D_2_ receptors contributes to the marked weight gain associated with the use of atypical antipsychotic agents^35^.

In Parkinson’s disease there is a substantial loss of dopamine-synthesizing neurons in the VTA, as well as the substantia nigra^36–39^. It is, therefore, interesting to note that patients with Parkinson’s disease have weight loss associated with an increased basal metabolic rate^40–42^. This elevated energy expenditure is reduced by dopamine-replacement therapy^43^ and by the use of therapeutic brain stimulation^44^. Apomorphine (dopamine D_2_ agonist) infusion for amelioration of Parkinson’s disease symptomology also leads to weight gain^45^. Patients with Parkinson’s disease are also reported to have higher brain temperatures and lower hand temperatures, consistent with increased heat production and/or decreased heat loss^46–48^. This could be related to loss of dopamine neurons in the VTA.

## Conclusion

Quinpirole-mediated stimulation of central dopamine D_2_ receptors reduces the BAT thermogenesis that contributes to emotional hyperthermia. The brain neural circuitry mediating this response includes the indirect pathway linking the LHb with the medullary raphé - a pathway that may include the dopamine-synthesizing neurons of the VTA. Quinpirole’s inhibition of emotional hyperthermia could be mediated via the actions of VTA dopamine neurons, or their rostrally-innervated target nuclei.

## Data Availability

The datasets generated during and/or analysed during the current study are available from the corresponding author on reasonable request.
